# Beriberi in Brazil: the historical constitution of a disease

**DOI:** 10.1590/S0104-59702026000100022en

**Published:** 2026-07-24

**Authors:** Sônia Maria de Magalhães

**Affiliations:** i Faculdade de História Universidade Federal de Goiás Goiânia GO Brazil soniademagalhaes@ufg.br Professor, Faculdade de História/Universidade Federal de Goiás. Goiânia – GO – Brazil. soniademagalhaes@ufg.br

**Keywords:** Beriberi, Science, Diet, Vitamin, Hunger

## Abstract

Deficiency diseases were first investigated in Brazil in the second half of the nineteenth century, especially in response to reports of urban poverty. Beriberi, which is currently understood as resulting from vitamin B1 deficiency in the human body, is one of the diseases most researched by scientists and doctors. This analysis proposes to historicize the disease by revisiting the medical debate, observing its social impacts until the constitution of a medical-scientific fact, when Brazilian doctors, inspired by infection- and nutrition-related theories, engaged intensively in discussions until a thought collective was formed, as conceived by Ludwig Fleck, and they agreed it was a vitamin deficiency.

## Historicizing beriberi

Research on beriberi in Brazil, aimed at developing a conception of the disease, must take a historical approach, as the Polish physician and biologist Ludwig Fleck (2010) so rightly sustained in the 1930s. This conception paved the way not only for the development of the humanities but also for disease to be understood as a sociopolitical and intellectual construct in the field of science. This perspective was heralded by Fleck when, writing about the genesis and development of a scientific fact, he stated that a conception of syphilis could not be reached unless a historical perspective was taken. In his view, the discovery of the etiological agent alone was not enough to determine a disease, since the concept of nosological entity takes shape in a historical process. As such, technical progress should be perceived not as an isolated event but as the result of experiences produced over time. In Fleck’s view, a disease, like beriberi, is a human and historical event; thus, its social and cultural understanding will change as science itself progresses.

The medical term “beriberi” comes from the Sinhalese word for weakness. It has been known since ancient times in Japan and is also described in early Chinese medical treatises. As to Brazil, there is no specific time that could be identified as the beginnings of the disease in the territory. Before it was identified as such, it was referred to as “swelling of the legs” and “leg pads” (*perneiras*), among other things. In 1648, a Dutch physician, Guilherme Piso (1957), recorded an ailment much like beriberi that he called “stupor of the limbs.” According to Almeida (1916), in 1756, the paralysis that was endemic in Maranhão (northeastern Brazil) could have been the disease. In Rio de Janeiro, in 1786, there were reports of an epidemic called *zamparina*; in the captaincy of Rio Negro, Antônio José Braga made reference to cases of *paralysia beriberium*.

Beriberi was endemic in nineteenth-century Brazil, and sometimes there were outbreaks of epidemics. This prompted physicians of the day to start to map its spread in different regions, in what would today be termed medical geography (Loureiro, 1885). In 1866, an epidemic broke out in Bahia, as researched and reported by Doctor José Francisco da Silva Lima, in whose opinion, beriberi, which affected all individuals regardless of social status, was marked by numbness of the extremities, skin irritation, general weakness, and other symptoms, and could be manifested endemically or epidemically. He published his findings in several issues of *Gazeta Médica da Bahia* under the title “Contribution to the history of a disease that currently reigns in Bahia in epidemic form and is characterized by paralysis, edema, and general weakness.” The articles that appeared in the journal, published in the northeastern province of Bahia, were subsequently compiled by José Francisco da Silva Lima in *Ensaio sobre o beribéri no Brasil* (Essay on beriberi in Brazil). Lima (1872) reported that the epidemic had devastated several parts of the province and affected all social classes. He described the clinical process with a wealth of observations and classified beriberi into three clinical forms: paralytic, edematous, and mixed. His meticulous investigation included autopsies on enslaved Africans who died in prison and were subject to police examination, through which he found that the disease did not seem to spread through contagion or infection, but rather due to unidentified general hygienic conditions (Lopes Filho, 1998). Almeida (1916) later praised Silva Lima for his work, stating that he had laid the foundations for the dietary theory that gained traction in the early 1900s.

In Mato Grosso, central west Brazil, according to the Viscount of Taunay, the retreat from Laguna (1867), after the conflict with Paraguay, was ravaged by beriberi: “Beriberi continued to claim numerous victims in our ranks in that place still subject to the influence of the great swamps that the troops had just crossed, between Coxim and Miranda” (Taunay, 2011, p.37). The viscount interpreted the event as a new type of climatic epidemic, reflex paralysis, or beriberi, which decimated even more soldiers.

In a memoir entitled “Beriberi in the province of Minas Gerais,” dated 1872, Antônio Felício dos Santos (1875) discusses the incidence of the sickness in the region, which had first appeared at the Seminary of Our Lady of the Good Death (Seminário de Nossa Senhora da Boa Morte) in Mariana (1858-1861) and had later spread to the School of Caraça (1861),^1^ Diamantina (1876), and Patrocínio (1876) (Azevedo, 1875). Santos conducted a thorough examination of his patients: he included their age, he observed the progression of the disease and at what time of year it occurred, and he also listed geographical, environmental, and epidemiological information to formulate the nosological circumstances of the cases. According to Lopes Filho (1998), while Felício dos Santos and Silva Lima may not have categorically stated that a microbial agent was responsible for beriberi, they did not reject this idea either, with such considerations facilitating the spread of germ theory.

In the province of São Paulo, southeastern Brazil, beriberi was studied by the Italian physician Ignazio Aquiles Betoldi, who was adamant in affirming its existence in the region. In so doing, he was apparently contradicting many of his colleagues, who he claimed denied its presence: “The vast majority of the practitioners in the province deny its existence and say that it only exists in our wild imagination, for which all diseases are beriberi” (Betoldi, 1878, p.3). As for its etiology, he believed it was caused by a miasma, like typhus, intermittent fever, yellow fever, and all infectious diseases. However, he continued, “it is a *sui generis* miasma, because its effects are also *sui generis*” (p.9). He also warned “that beriberi cannot be an offspring of malaria, because it occurs where there is no standing water, as in the high and airy mountains of Minas Gerais” (p.11). This passage is interesting because it shows that he had access to the research conducted by Felício dos Santos in Minas Gerais. Beriberi continued to be associated with malaria until the early years of the twentieth century. Yet in the Amazon, studies by Carlos Chagas (1972) ruled out this idea, finding that many cases attributed to beriberi were a type of quartan malaria or a more virulent variety.

In 1888, Virgílio de Mello Franco recorded the incidence of beriberi in Goiás, describing it as a disease known as “swelling of the legs” found almost everywhere in Brazil, but particularly so in the northern provinces and in inland Goiás and Mato Grosso. He lamented the delay in the arrival of empirical medicine to the inland regions and the irrational treatments used, especially for this disease, which he believed to be characterized by severe anemia (Franco, 1888). Death records issued by the St. Peter of Alcântara Charity Hospital (Hospital de Caridade São Pedro de Alcântara) in the city of Goiás, Goiânia, confirmed that the ailment had spread throughout the region in the nineteenth century (Magalhães, 2014b).

In 1899, a German physician, August Hirsch, recorded the uncontrolled spread of the disease in Brazil, especially in Amazonas, Pará, Maranhão, Bahia, Espírito Santo, Rio de Janeiro, São Paulo, Paraná, Santa Catarina, and Mato Grosso (Kiple, 1988).

The population growth in Rio de Janeiro, Salvador, Recife, São Luís, and São Paulo in the eighteenth and nineteenth centuries brought social issues to the fore, prompting studies into poverty, disease, and malnutrition (Magalhães, 2014b). Nineteenth-century medical monographs began to focus on topics related to nutrition, housing, customs, and health, observing the relationship between these factors and the occurrence of endemic and epidemic diseases. Manuel da Gama Lobo (1865) linked xerophthalmia (corneal damage) and hemeralopia (night blindness). among enslaved Africans to a nutrient-poor diet, consisting of cornmeal porridge, pumpkin, and dried meat or bacon once or twice a week. In *O regime alimentar no Norte do Brasil* (The diet in northern Brazil), published in 1881, Raimundo Nina Rodrigues pointed to nutritional deficiencies resulting from a diet restricted to cassava flour.^2^ In 1887, Hernani da Silva Pereira, a graduate of the Bahia Faculty of Medicine (Faculdade de Medicina da Bahia), reported on how people’s diets varied according to their level of wealth, with the wealthiest enjoying fresh meat and beans, and the poorest having a monotonous diet based on dried meat, cornmeal, and cassava flour (Vasconcelos, 2007).

It is worth noting that studies conducted by doctors in Bahia in the field of food hygiene were the first to produce compelling reports of beriberi, long before the scientific discovery of vitamin B1.

Even though medical reports show that beriberi was widespread in nineteenth-century Brazil, there was no legal requirement for it to be reported to the competent authorities. This explains, at least in part, the dearth of information from some regions.^3^ The health legislation legitimized the established scientific knowledge by recognizing the existence of an infectious agent that caused beriberi (Lopes Filho, 1998). In 1889, new public health legislation was passed that made its notification optional (Brasil, 18 dez. 1889). It was only in 1904, with the passing of Decree 5,156 (Brasil, 8 mar. 1904), that the reporting of cases of beriberi was made mandatory. This in turn resulted in more detailed investigations of cases, assessments of the risks people were exposed to, recording of cases by geographical area, and definition of priority measures.

At the Bahia Faculty of Medicine, beriberi attracted particular attention between 1877 and 1921, when its origins were discussed in at least 19 theses (Meirelles et al., 2004), albeit without any definitive conclusion being reached. Food and vitamin deficiency were also popular research topics, accounting for seven theses. However, the etiology of the disease remained a mystery (Magalhães, 2014a). Nonetheless, in 1904, Nina Rodrigues, from Bahia, investigating cases of deaths at the St. John of God Asylum (Asilo São João de Deus), hypothesized that the causes of the disease there should be sought in the abject living conditions of the patients, who were mistreated and underfed. In other words, he cogitated the idea that the disease’s etiology was associated with some deficiency, not a miasma, and was not spread by contagion (Jacobina, Carvalho, 2001).

Research into beriberi by Brazilian and foreign doctors intensified as of 1880. K.C. Carter (1977, p.125) quantified the remarkable interest in the subject based on literature reviews conducted by Henrich Botto Scheube and William Leonard Braddon:

Scheube’s nearly complete bibliography includes two publications on beriberi between 1800 and 1809; in subsequent decades the publications numbered eight, ten, eleven, thirty, sixty-four, eighty, and, between 1880 and 1889, one hundred and eighty-one. W. Leonard Braddon’s incomplete bibliography lists nearly two hundred articles and books for the decade from 1890 to 1899; another incomplete bibliography lists two hundred and fifty publications for the period from 1900 to 1910. A complete bibliography for this period alone would certainly approach five hundred items.

Carter (1977, p.125) mentions that three decades later, in 1911, an editorial in the *Lancet* reported that “there is probably no disease concerning which so much discussion as to its etiology has taken place as beriberi.”

*O Brasil Médico* is also a prime source for the historical debate on the etiology of beriberi from 1885 to the mid-1950s,^4^ when coverage of the disease ceased to be a priority for the journal’s contributors. However, at the same time, studies on vitamins were gaining increasing publication space. Overall, a total of 99 articles on beriberi were published in *Anais da Academia de Medicina do Rio de Janeiro* from 1885 to 1887 and *O Brasil Médico* from 1888 to 1942.

**Figure 1:**
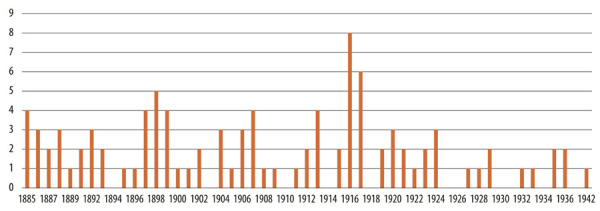
: Graph showing the number of medical articles on beriberi in the journal *O Brasil Médico*, 1888-1942 (source: made by Sônia Maria Magalhães)

A literature review of the database of the National Library (Biblioteca Nacional) retrieved 64 works on beriberi. Taken together, the publications in *Gazeta Médica da Bahia* (Meirelles et al., 2004), *O Brasil Médico,* the National Library archives, and the Fiocruz archives reveal how vigorous and prolific a debate the subject inspired, in line with discussions taking place internationally.

Since the nineteenth century, when the etiology of beriberi began to be intensively investigated, there had been no consensus in medicine about its origin, and its mode of transmission was not known. At this point, the hypotheses on the table were: infection by deadly miasmas – harmful emanations from the environment – or person-to-person transmission of a specific poison via objects or the air breathed by the sick (Chalhoub, 1996, p.64). Subsequently, these theories were adapted to take account of new scientific developments and germ theory, as developed by Louis Pasteur and Robert Koch, which were understood to be responsible for a number of different diseases. These new developments inspired major changes to the therapeutic and prophylactic methods and procedures for treating diseases. However, “their premises were slow to penetrate medical knowledge and practice, and they also sparked various disputes and controversies between supporters and opponents of this new paradigm” (Malaquias, 2016, p.736).

As the theory of nutritional deficiency seemed incompatible with the facts, most early observers tended to support other explanations for beriberi. Arguably, the most common of these were the miasma and the malaria theories. One of the first westerners to investigate the disease in Japan, William Anderson, noted that most Japanese doctors believed it was caused by some poisonous emanation from the soil. Accordingly, he concluded that there was probably an atmospheric “poison” that caused it (Carter, 1977). In a similar vein, Doctor Betoldi (1878) believed that the disease prevalent in the province of São Paulo was caused by miasmas.

Just as there were those who defended the theory of infection, there were others who drew on recent research linking the origin of the disease to nutritional deficiency. In 1889, the Dutch researcher Christiaan Eijkman (1858-1930) isolated a water- and ethanol-soluble substance from rice skin, which he called the antineuritic principle, claiming that it cured polyneuritis in birds (Rezende, 2009). Taking the theory further, another Dutchman, Gerrut Gerrit Grijns (1865-1944), formulated the theory that the disease was not caused by a toxin in unpolished rice but by not consuming a substance in the rice skin, which is removed in the rice polishing process (Rezende, 2009).

In 1910, various research endeavors quickly converged on the idea that the culprit was nutritional deficiency. According to Carter (1977), epidemiological studies (Takaki, Voderman, Braddon, Fletcher, Stanton, and Fraser) and animal experiments (Eijkman, Grijns, Holst, and Frolich) showed a connection between certain diets of ground grains and various disorders. Some chemists (Eijkman, Fraser and Stanton, and Funk) later isolated and characterized with great precision the specific element in rice polishings that might prevent or cure some of these disorders.

The graphic in Figure 1 shows how the medical debate intensified in the second decade of the twentieth century, especially in 1916, thanks to the spread of the theory of vitamins, first formulated in 1911 as “vitamine” by the Polish chemist Casimir Funk,^5^ who identified their chemistry after studying thiamine, later called vitamin B1. Funk’s discoveries sparked a heated debate about beriberi among Brazilian doctors. Subsequent issues of *O Brasil Médico* reveal how discussions about vitamins gained increasing traction in the beriberi debate.

In the same year as the upsurge in publications on the subject in *O Brasil Médico*, a Minas Gerais-based physician, Theophilo de Almeida, who supported the dietary theory, defended his thesis entitled *O beribéri no Brasil* (Beriberi in Brazil). In it, he was critical of his colleagues’ lack of interest in the disease, pointing out:

Recently, a long discussion has broken out within the ‘National Academy of Medicine’ and in the press, reviving old etiological theories, multiple pathological conceptions, raising a veritable scientific uproar whose great fruit, if not reaped by others, has undoubtedly been to draw the attention of Brazilian doctors, who were already, unfortunately, forgetting their ‘beloved’ beriberi (Almeida, 1916, p.22).

Almeida (1916) then stresses that the only studies worth mentioning were the ones by Antônio Austregilo Rodrigues Lima, Oscar de Souza, Henrique Duque and João Soledade, J. Silvado, Júlio Novaes, and Lídio Parahyba. He further reflects that the polished rice theory needed more in-depth study in Brazil.

### The Brazilian diet

As of the 1920s, the link between beriberi and nutritional deficiency was widely reported in the pages of Brazil’s leading newspapers. Their readership gained increasing access to the debate among Brazilian scientists thanks to the circulation of ideas, exchanges, contacts, distributions, connections, assimilations, and reinterpretations surrounding the subject. As Ilana Löwy (2012, p.24) points out, “‘scientific facts’ were also enriched by their circulation outside the restricted domain of scientific research. Fleck was thus interested in the interactions between scientific and popular representations of natural phenomena.” As for how this occurred, Kapil Raj (2015) notes that the concept of circulation highlights the power relations, resistances, and negotiations inherent to all intercultural interactions. His studies reveal the existence of medical and scientific communities in Brazil geared towards solving the country’s problems, casting into question the leadership of a science developed in line with models from abroad, especially the United States and Europe. This dynamic process allowed different actors involved in the scientific debate greater or lesser agency. In a front-page interview for the newspaper *A Noite*, published on August 23, 1924, the physician José Paranhos Fontenelle criticized the Brazilian diet of meat, beans, and rice and noted evidence of more regular consumption of fruits, vegetables, and milk.^6^ He also made notable reference to the development of science and the discovery of vitamins:

Another very important modern finding in food science was the demonstration of the need for certain substances that are indispensable for Nutrition in order for growth to be accomplished and health to be maintained, substances that exist in very small quantities, but which have great power to stimulate and regulate nutrition, which are known as ‘vitamins,’ existing in relatively high concentrations in milk, vegetables, fruits, and cod liver oil. These vitamins are already classified into several groups, and a lack of some of them causes beriberi, scurvy, rickets, poor growth, anemia etc. (Fontenelle, 23 ago. 1924).

As for the eating habits of Brazilians, he highlighted how little milk they consumed and recommended its inclusion in the daily diet. This issue became a matter of state in the 1930s, when major investments were made in advertising for milk, in view of the importance attached to it by the new science of nutrition; however, there were obstacles of various kinds to its dissemination. According to Brinkmann (2014), it was very hard to ensure a supply of cow’s milk in Rio de Janeiro, as not only would a cultural change in eating habits have to be achieved, but a supply system would have to be introduced that at the time exceeded the Brazilian state’s capacity. In addition, milk was expensive for most people, making it a luxury rather than a necessity. Fontenelle (23 ago. 1924) reserved a special place for women, warning of the need to “intensively promote the value of various foods and teach housewives different ways to vary their diets, enriching Brazilian cuisine with dishes that feature milk and vegetables. Everything must be done to combat the meat-rice-beans system, whose elements are excellent, but do not suffice on their own.”

Even with the communication of new experiments supporting the dietary etiology of beriberi, the Brazilian medical community had still not reached a consensus on the matter by the mid-1900s. According to Fleck, developing a thought collective depends on having a community whose members influence each other with ideas and shared knowledge, building a body of knowledge oriented toward understanding and changing the status quo as a result of social – and thus collective – activity.

In the 1930s, the problem of the etiopathogenesis of beriberi still divided doctors in Brazil into two groups: the pro-vitamin camp and the pro-infection camp. Table 1 shows the supporters of each of these two positions.

**Table 1:** : Supporters of the two rival theories about beriberi

Infection theory	Vitamin theory
Silva Lima, Torres Homem, Martins Costa, Pacífico Pereira, Francisco de Castro, Azevedo Sodré, Pedro de Almeida Magalhães, Sampaio Vianna, Miguel Couto, Clementino Fraga, Oscar de Souza, Eduardo Meirelles	Arlindo de Assis, Afrânio Peixoto, Hélio de Araújo Maia, Vicente Baptista, Figueiredo Rodrigues, Theophilo de Almeida

Source: Peregrino Jr. (1935).

Curiously, Peregrino Júnior brought back into play and even validated studies from the nineteenth century, suggesting that beriberi may be caused by microorganisms found in patients (Almeida, 1916, p.21-22). Pacífico Pereira had found some round corpuscles in the blood of patients in Bahia; Augusto Maia had discovered a type of micrococcus; Paulo Mendes had observed some germs in bone marrow. In 1883, João Batista de Lacerda had come across a bacillus he believed to be the cause of the disease. And in 1898, Francisco Fajardo had detected a hematozoan similar to that of malaria (Almeida, 1916). According to Carter (1977), germ theory influenced many scientists at the time and fostered demographic studies and chemical experiments on animals that opened the way for the theoretical understanding of the disease. So it was that science moved forward in pace with the experimental testing of all the hypotheses – whether they were valid or not.

In a text from 1913, “Notes on the epidemiology of the Amazon,” Carlos Chagas (1972, p.167) comments that the severe beriberi epidemics and the many cases of polyneuritis (multiple nerve inflammation) in Acre shared edema as a common trait, but that the patients did not display the characteristic disturbances of the disease, which he identified as “forms of malaria.” He confirmed the rarity of the cases and referred to an especially deadly condition, which he called “galloping beriberi,” in which edema began in the lower limbs and then spread up the trunk, leading to death in a short period of time.

Afrânio Peixoto (1972), in turn, attested that beriberi in the Amazon was caused by nutritional deficiency: vitamin-poor diets based on cassava flour. To prevent and treat it, he recommended improving the diet, including eggs, fish, beans, and fresh fruit and vegetables.

Theophilo de Almeida (1916, p.19) observed the repercussions of the studies supporting the nutritional theory: “Rice has gained prestige in the etiology of beriberi, despite the naysayers, even in the Orient, the International Congress of Medicine held in London in 1913, adapting Braddon’s proposal,^7^ passed the following resolution: it recognizes that a diet based on the continuous consumption of the grain in the form of white rice causes beriberi.”

Despite opposition, the 1913 International Congress of Medicine had confirmed the dietary theory based on the consumption of polished white rice and ruled out the theory that beriberi was an infection. The London declaration basically endorsed the hypothesis of a British doctor, William Braddon, who observed the pathogenic origin of the disease, attributing it to a “poison” (produced by an epiphytic or parasitic organism) present in rice (Almeida, 1916). The event thus also recommended stopping using quarantine to control beriberi. As for how well the dietary theory was accepted by the Brazilian delegates Juliano Moreira and Ernesto Crissiuma, Almeida states that they did not take part in the discussion.

Afterwards, Crissiuma gave an interview to *A Noite* newspaper:

As for beriberi, another order of the day was voted on that called for the abolition of quarantine for ships coming from countries infected with the disease, as beriberi is not considered contagious, and also the prohibition of white rice as the staple for the working classes, especially the ‘collies’ [*sic*], who work in India and China (Entrevista..., 6 dez. 1913).

Crissiuma admitted that the congress, which had attracted some eight thousand attendees, had produced great results: “This time I can assure you that the congress participants worked seriously on the most pressing issues that are of greatest interest to science in each of the countries represented there” (Entrevista..., 6 dez. 1913). In addition to beriberi, syphilis, leprosy, and leishmaniasis were widely discussed, featuring prominently in the clinical debates of France, the United Kingdom, and Germany. Apart from Moreira and Crissiuma, the Brazilian delegation was also comprised of Clementino Fraga, Marcos Cavalcanti, Henrique Roxo, and Rocha Lima.

The theory that polished rice was the cause of beriberi sparked controversy among Brazilian doctors, since it did not explain the incidence of the disease in the country: polished rice was not a key element of the diet except in some northeastern areas. Fresh beef was available, but it was expensive and was not widely consumed. In Rio de Janeiro and São Paulo, poor people only had access to dried meat. The meat-drying process, in which meat was first salted and then dried in the sun for at least two months, actually destroyed the thiamine in the beef. As it is highly water-soluble, most of it was discarded in the water used to soak the meat. The rest of the thiamine would have been destroyed in the cooking water (Kiple, 1988).^8^

However, the biggest villain in the Brazilian diet was the dependence on cassava flour, which contains even less thiamine than white rice. In northeastern Brazil, where the flour was a staple of most people’s diet, beriberi was more harmful. The same thing occurred among the poor populations of São Paulo and Rio de Janeiro, whose diet was based largely on dried meat. In Kiple’s (1988) view, the food theory was justified in Brazil by the harmful combination of dried meat and cassava flour. However, it was only in the twentieth century, thanks to the emerging science of nutrition, that beriberi was proved to be a form of hypovitaminosis.

In the 1930s, there was still no consensus amongst medical practitioners as to whether beriberi was caused by vitamin deficiency. In an article entitled “Vitamins and avitaminosis,” published in *O Brasil Médico* in 1935, Oscar Ferreira Júnior, assistant to Oswaldo de Oliveira, the first full professor of clinical medicine, wrote: “The problem of vitamins in the etiology of the disease is by no means resolved; the problem remains complex and intractable” (Ferreira Jr., 20 jul. 1935, p.654).

Brazilian researchers who supported infection- and diet-related theories worked hard to describe beriberi and its symptoms, establishing its clinical characteristics. The debate surrounding its causes and origin resulted in the establishment of a specialized medical field in the area. Accordingly, medical statements gained social relevance as the research results were communicated in newspaper editorials, conferences, and other media, describing the symptoms, researchers’ perceptions, and scientific narratives. However, in the medical community, controversy still abounded. Due to the limitations of medicine and the difficulty of making a reliable clinical diagnosis of beriberi at a time when there were no laboratory techniques for this process and microscopes were not widely available, diagnosing beriberi was a stumbling block even for doctors. Essentially, in the face of limited knowledge about the ways the disease presented, its prevalence in some regions, and the lack of knowledge about it in others, medical diagnoses were often inaccurate or simply wrong.

The contributions of the research carried out generated wide debate around beriberi and provided essential inputs for framing the questions that needed to be answered. As Fleck (2010, p.153) explains,

each fact has a retroactive impact on others, and each change, each discovery, has an effect on a field that is actually limitless: in knowledge developed in the form of a harmonious system, each new fact alters all previous ones, however small that alteration may be. In this case, each discovery is actually the recreation of the whole world of a thought collective.

Various relatively independent avenues of research formed the basis for the endorsement of the deficiency theory. However, they had to be organized and made coherent. The first publication to bring together the different strands was Casimir Funk’s 1912 text “The etiology of the deficiency diseases,” where he presented a new substance that was essential for the maintenance of life: the “vitamine” (Carter, 1977).

All of this constituted the ongoing development of a thought style, as conceived by Fleck. Beriberi paved the way for a new interpretation in the field of medicine, where the idea that it was an infectious disease was discarded as it was gradually understood from a new perspective, that of nutrition – an understanding that came to be shared by researchers, albeit only after decades. It was thus constituted as a medical-scientific fact, since it was assimilated and expressed in the thought collective that was itself taking shape at the time.

### Vitamins at the center of the debate

With the spread of the theory of vitamins, the inadequacy of the Brazilian diet – poor in milk, dairy produce, eggs, vegetables, and fruits – was put on the public agenda. This coincided with a growing recognition of nutrition as a science, at a time when new knowledge about food composition and the connections between diet and disease called for action of specialized professionals (Magalhães, 2014b, p.44):

As progress was made in biochemistry, which led to a better understanding of the functioning of the human body and the identification of new substances, like vitamins, this topic became the target of investigations, studies, and inquiries by scholars from different areas of knowledge.

It was in this vibrant context, in the 1930s and 1940s, that two nutritionists, Josué de Castro and Nelson Chaves, conducted some important studies on Brazilian foods. Their work coincided with a time when the issue of hunger – exacerbated by the rising cost of living in the wake of the 1930 political crisis – was galvanizing various sectors of Brazilian society (Magalhães, 2014b, p.44). According to Leme (2021, p.1127), “the new scientific perspective therefore provided a key tool for Castro and his peers to look at the food issue in Brazil – the calorie paradigm.”

In 1932, Castro conducted a pioneering food survey on the living conditions of workers in Recife,^9^ northeastern Brazil, which revealed how poor the diet of this population was, as well as the marked calorie deficit. This study was in line with a growing body of research in the early twentieth century that aimed to quantify and assess the diets of Europe’s working classes, taking advantage of the recently acquired ability to measure foodstuffs (Leme, 2021). Castro’s work revealed the great evil that was afflicting workers in the Northeast: hunger was what prevented them from keeping pace with workers in the south of the country, or even in the inland region, which he identified as having better eating habits. His work laid the blame for the northeasterners’ reduced productivity and, indeed, most of their illnesses and deaths, squarely with the phenomenon of chronic hunger, which this population had endured for centuries.

In *Geografia da fome* (Geography of hunger), Castro (1948) mapped out the food shortages in different regions of Brazil, namely, the Amazon (Amazônica), the Sugar-Producing Northeast (Nordeste Açucareiro), the Inland Northeast (Sertão Nordestino), the Central West (Centro Oeste), and the Far South (Extremo Sul).

**Figure 2:**
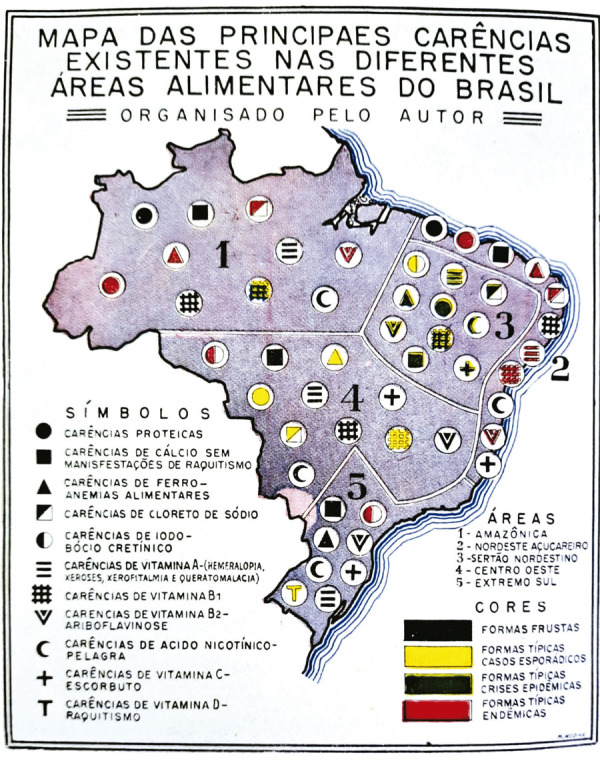
: Map of the main dietary deficits in different areas of Brazil (source: Castro, 1948, Figure 6)

Castro identified clear signs of hunger in three of the five areas he analyzed: the Amazon, the Sugar-Producing Northeast, and the Inland Northeast. In contrast, the Central West and Far South did not exhibit such notable deficiencies, even if their diet was not ideal. Castro’s map shows how widespread beriberi was in areas now classified as the North and Northeast and, to a lesser extent, in the South and Southeast. According to Kiple (1988), beriberi was most prevalent in the Northeast, where people’s basic diet – a mixture of dried meat and cassava flour – was deficient in vitamin B. According to Araújo (2022), diseases related to nutrient deficiencies in Rio Grande do Norte (northeastern Brazil) were caused by the inequality between the large landowning oligarchs and the people who worked on their estates. It is important to note that Castro supported the dietary theory of Brazilian beriberi, caused by the diet of dried meat and cassava flour. In Kiple’s (1988) opinion, in Minas Gerais and Rio Grande do Sul (southeastern and southern Brazil) there was less variation in the diet consumed by the rich and poor. In the former, all social groups regularly consumed fresh pork, beans, and cornmeal. While corn was deficient in thiamine, fresh pork was rich in niacin, making up the required vitamin B intake. As for the latter group, they regularly consumed fresh beef, cereals, and vegetables. Interestingly, while they also produced dried meat, they did not consume it regularly but sold it to northeasterners.

Castro and Chaves both reaffirm the emphasis on diet over the race/climate dimension in their explanations for Brazilians’ quality of life. In *Alimentação e saúde pública* (Food and public health), Chaves (1948) blames malnutrition for the high rates of infant mortality, stillbirths, childhood tooth decay, stunted growth, deficiency diseases, beriberi, pellagra, and parasitic worms. Elements of the theory of the vicious circle of poverty and disease, as identified by other contemporary authors, are also present in Chaves’s studies: “Man produces little because he has a poor diet and is sick, and because of his educational level he has very limited set of aspirations, and because he produces little he does not have enough food” (cited in Vasconcelos, 2001, p.329). In Pernambuco (northeastern Brazil), attention was drawn to the problem of monoculture and its repercussions, especially on the nutritional status of the people of the Zona da Mata region. In Castro’s opinion, cassava flour with beans, dried beef, and coffee sweetened with sugar were gradually losing their place on the agricultural workers’ meal table. Instead, their diet comprised no more than cassava flour and watered-down beans. Chaves’s (1946, 1948) research drew the attention of the authorities to the high incidence of dwarfism in the Zona da Mata region of Pernambuco. In his view, people’s stature and even their brains were gradually dwindling due to hunger. Drawing on the themes of hunger and eugenics, Pernambuco’s scientists contributed to the nation-building enterprise as they endeavored to shift their analyses from racial to sociocultural considerations, with the racial/climatic prejudice that existed in relation to miscegenation (Magalhães, 2014b). Supporting these arguments, Lima (2008, p.265) notes that “what united intellectuals around the demonstration of the hunger disease thesis between 1934 and 1939 was the prospect of eugenic improvement as a factor of evolution.”

At that time, food shortage was already widely discussed in the Brazilian press and by experts. Among them, the work of Castro and Chaves – both professors and researchers at the Recife Faculty of Medicine – gained prominence in the context of national and international research on diet, hunger, poverty, and nutrition. Indeed, from that time on, they were supported in the legitimization and communication of nutrition-related theories through the intermediation of state policies. Chaves held the positions of director general of the Department of Public Health and secretary of Health of Pernambuco. As for Castro, he became involved in the first vitamin campaign carried out in Brazil, which was covered extensively in *A Noite*: “As previously reported in the press, the Economic Mobilization coordination team has resolved to launch the National Vitamin Campaign, which aims to provide the Brazilian population with these elements, which are so necessary for life. The campaign will be run by a steering committee, chaired by Minister João Alberto, and whose members include Messrs. Josué de Castro, head of the Technical Food Service C.M.E.” (A Noite, 14 jan. 1944). Thus, from 1934 to 1939, the Brazilian state apparatus promoted political and pedagogic actions in the realm of nutrition education, helping foster an understanding of how to eat well and make the most of the household budget (Lima, 2008).

Doctor Theóphilo de Almeida was also indirectly involved in state recognition of the nutritional theory of beriberi through his roles in public office. In 1923, he started working as a physician with the National Department of Public Health (Departamento Nacional de Saúde Pública), headed by Carlos Chagas. In 1941, the decade in which the vitamin campaign began, he began serving as director of the Hospital Division in the Ministry of Health (Lopes Filho, 1998).

One group of professionals who played a key role in the quest to discover the etiology of beriberi and ways to combat it was the group researchers, mostly physicians specialized in nutrition, working on state projects. They undertook specialized research, which enabled them to form a select “esoteric” field, which was responsible for developing a thought collective around the disease. However, the understanding of beriberi extended to other domains, as a broad field of knowledge grew up around this group, composed of nonspecialists (whom Ludwig Fleck calls “exoteric”) working in different press outlets, such as journalists from *A Noite*, who took on the task of passing the knowledge produced by specialists on to their readers. By reproducing their ideas, these nonspecialists endorsed the guidelines passed down by the “esoteric” collective, validating and expanding the reach of their ideas, which goes to show the interdependence of these fields for the social legitimacy of science (Fleck, 2010, p.26). By simplifying the clinical discourse, making it accessible to the lay public, and demonstrating the impact of the disease on the health of the population, they certified the “esoteric” discourse by spreading their conceptions about the disease.

The success of the pedagogical work done by the *exoteric* collective is attested, for example, in a piece published in *A Noite* on March 27, 1953, under the headline “Pondo os ‘pontos nos ii’” (Dotting the i’s). The piece in question was a quiz that readers could play with their friends. There are two columns, one containing the question and the other, the answer. The fourth question is: “Does eating rice cause the disease called beriberi?” And the answer: “Beriberi is not caused by eating rice, but by an unbalanced diet or lack of vitamin B, which happens from eating only polished rice” (A Noite, 27 mar. 1953, p.7).

After studying the disease for decades without uncovering its etiology, now researchers began to conduct dietary research that allowed them to develop narratives in the scientific field, describing the geography of the disease, the living conditions of the population, biological and sociocultural characteristics, and dietary options, thus consolidating a way of conceiving it, along with other vitamin deficiency diseases, in the field of medical science. However, Castro was not the only one to do such surveys. Leme (2021) mentions research commissioned by the São Paulo Institute of Hygiene (Instituto de Higiene de São Paulo), published in 1935, whose lead researcher was Geraldo Horácio de Paula Souza, in partnership with Ulhôa Cintra and Pedro Egídio de Carvalho. According to Fleck, these surveys consolidated a particular though style. Their findings and the ideas they contained were gradually spread among specialists and enlightened laypersons through associations, medical conferences, scientific events, interviews, and newspaper editorials, creating a common perception among these stakeholders. Through these actions, the prevailing conceptions of beriberi were spread and consolidated, but also, even in the field of medicine, contested and revised.

## Final considerations

The controversies surrounding beriberi and germ theory in the late 1800s and nutritional deficiency theory in the early 1900s provide an opportunity to retrace the institutionalization of science in Brazil. Germ theory was a new conceptual prism through which to understand beriberi as being related to nutritional deficiency. However, the road towards constructing a medical consensus, or thought collective, as conceived by Fleck, around the idea that it was caused by vitamin B1 deficiency was long and contested, since there was such a wide range of competing ideas about its etiology. For example, beriberi was often observed in societies where rice was a staple, which meant it could be related to the deterioration or contamination of the grain. It could have been associated with the intestinal worms found in some patients, which would give it a parasitic cause. It also abounded in specific environments, such as prisons, hospitals, schools, barracks, asylums, and ships, affecting many people simultaneously, which could indicate some type of contagion. From the 1880s onwards, several researchers (in Brazil and abroad) began to consider the possibility that microorganisms may be the cause. But it was only at the beginning of the twentieth century that the new proposition of nutritional deficiency was developed, when Casimir Funk attributed beriberi to the combined deficiency of several nutrients. In Brazil, the germ and vitamin theories coexisted well into the 1900s, prompting intense and lengthy debate among doctors. In this process, and in the wake of further research that supported Funk’s original claim, a thought collective emerged that recognized vitamin deficiency as the cause of beriberi.

Now, in the twenty-first century, beriberi is once again on Brazil’s medical agenda. In 2007, 47 people died of the disease in Maranhão. From a medical perspective, these cases constituted an outbreak of a disease that was understood to be under control. In 2008, suspected cases were identified among the Ingaricó and Macuxi indigenous groups in Uiramutã, a municipality in the Amazonian state of Roraima, and since then work has been done in partnership with the state and municipality to investigate, monitor, prevent, and control the disease. In 2012, new cases were identified among the poorest population in the north of the country. In response to these contemporary episodes, the Ministry of Health organized the publication, in 2012, of a guide on beriberi (Brasil, 2012). The publication provides guidance for health professionals working in the public health system, including the subsystem for indigenous healthcare, designed to promote the adoption of measures to detect, prevent, and control the disease. From 2017 to 2020, hunger and poverty grew in the country on the back of an economic crisis, which was exacerbated by the covid-19 pandemic, resulting in an increase in the number of deaths from beriberi. Santos et al. (2023) conjecture that the reason for the continued increase in deaths from beriberi recorded in 2021 and 2022 was a result of the worsening social and economic crisis. Research conducted during the pandemic shows indicates that food insecurity increased even further at the time, especially among the poorest, who simultaneously face multiple vulnerabilities. A neglected disease associated with poverty, beriberi is not on the list of diseases subject to compulsory notification in Brazil, which makes it more likely for cases and deaths to be underreported. This in turn keeps the disease in the shadows, along with those affected by it and its societal impacts and implications. Research by the Brazilian Institute of Geography and Statistics (Instituto Brasileiro de Geografia e Estatística) found that 3.2 million Brazilian households were affected by hunger in 2023, opening the door to serious nutritional diseases. Shortage of food was experienced not only by the adults but also by the children and adolescents of these households, a situation defined by the institute as severe food insecurity (Ferreira, 25 abr. 2024).

Reflecting on the history and impacts of beriberi in Brazil, from times past until the present day, is paramount if we are to understand how people and institutions have responded to this disease, how they have formed public health policies on the issue, and how they have implemented these policies to address and control the disease.

## Data Availability

Not deposited in a data repository.

## NOTES

1 According to Lopes Filho (1998), the most devastating epidemics at Caraça, apart from the one in 1862, were the ones in 1873, 1878, 1901, 1906, and 1910. By 1930, beriberi was declared absent from the institution.

2
Carvalho (2025) encountered the results of this study in the journal *Pacotilha*, Maranhão, 1888, year 8, issues 154, 157, 158, 164, 167, and 188.

3 The health legislation of 1889 made it possible, but not mandatory, for cases of beriberi to be notified to the authorities. What was mandatory was smallpox vaccination in the first six months of life, disinfection of contaminated places and objects, isolation in hospital when a patient was unable to receive treatment at home, and notification of communicable diseases. The diseases for which notification was mandatory were yellow fever, cholera, plague, diphtheria, smallpox, scarlet fever, and measles. Along with beriberi, notification of tuberculosis, typhoid fever, and whooping cough was optional. Only with the 1904 directive was notification of beriberi made mandatory.

4 *O Brasil Médico* was a weekly publication that carried scientific, political, practical, and professional news of interest to the Brazilian medical community. It was founded in 1887 and was published until 1956 in Rio de Janeiro.

5 Casimir Funk was Polish. Born in Warsaw in 1884, he earned a doctorate in chemistry from the University of Bern in 1904. He worked as a biochemist at the Pasteur Institute, the Municipal Hospital of Wiesbaden, the University of Berlin, and the Lister Institute, and pursued further studies in Paris and Berlin. He also worked in laboratories in London, New York, Paris, and Warsaw. In 1915, he migrated to the United States. From 1923 to 1927, Funk was head of the National Institute of Hygiene in Poland. Back in the United States, he became president of the Funk Foundation for Medical Research. He died in New York in 1967. Funk is recognized as one of the first scientists to formulate the concept of vitamins. Every year, the US-based Kosciuszko Foundation awards the Casimir Funk Natural Sciences Award to Polish American scientists who excel in the field. To learn more about Funk, see Griminger (1972).

6 Here, Paranhos is certainly discussing the diet of the wealthier populations, since the poor continued to consume essentially dried meat and cassava flour, as noted by Hernani da Silva Pereira in 1887 (Vasconcelos, 2001).

7 [Note by Almeida (1916)] According to Braddon, in places where it is endemic, or during an epidemic, only those who eat rice are affected by beriberi.

8 Kiple argued that the B complex must be in balance in the human body. It is worth mentioning that sunlight also destroys riboflavin, so dried meat does not contain enough thiamine and riboflavin in relation to niacin, which would considerably increase the normal vitamin B requirements of the individual who ingested it.

9 For Leme, hygienists began to do surveys to ascertain the habits of Brazilians in the early twentieth century, but without any pretense of statistical representativeness. For example, in 1912 Arthur Neiva and Belisário Penna researched the health conditions of the populations in the semi-arid inland areas of Pernambuco, Piauí, and Goiás.
