# Supplementary material for: Beriberi in Brazil: the historical constitution of a disease
Source: Hist Cienc Saude Manguinhos. 2026 Jul 24;33:e2026022. doi: 10.1590/S0104-59702026000100022en (PMC13403529; doi:10.1590/S0104-59702026000100022en)
Supplement: Translation

# Translation: O beribéri no Brasil: a constituição histórica de uma doença

**DOI:** 10.1590/S0104-59702026000100022

**Authors:** Sônia Maria de Magalhães

**Affiliations:** i Brasil Professora, Faculdade de História/Universidade Federal de Goiás. Goiânia – GO – Brasil. soniademagalhaes@ufg.br

**Keywords:** Beribéri, Ciência, Alimentação, Vitamina, Fome

## Abstract

As doenças carenciais começaram a ser investigadas no Brasil na segunda metade do século XIX, sobretudo, com a denúncia da pobreza urbana. Dentre as enfermidades mais pesquisadas pelos cientistas e doutores, destaca-se o beribéri, reconhecido atualmente como deficiência de vitamina B1 no organismo humano. Esta análise se propõe a historicizar a moléstia, revisitando o debate médico, observando os impactos sociais da doença até a constituição de um fato médico-científico, quando médicos brasileiros guiados pelas vertentes teóricas infecciosa e alimentar travaram intensas discussões até a composição de um coletivo de pensamento, conceito elaborado por Ludwig Fleck, admitindo-a como uma avitaminose.

## Historicizando o beribéri

A investigação sobre o beribéri no Brasil, que se propõe chegar a um conceito de doença, requer uma abordagem histórica, como bem afirmou, na década de 1930, o médico e biólogo polonês Ludwig Fleck (2010). Essa compreensão possibilitou, além do desenvolvimento das ciências humanas, refletir a doença como construção sociopolítica e intelectual no meio científico. Tal perspectiva se tornou possível quando o cientista concedeu a gênese e o desenvolvimento de um fato científico em torno da sífilis, ao afirmar não ser possível chegar a um conceito da doença sem uma abordagem histórica. Na sua percepção, a descoberta do agente etiológico da enfermidade por si só não determinaria a moléstia, pois o conceito de entidade nosológica é decorrente de um processo histórico, considerando que o avanço técnico deve ser percebido não como um evento isolado, mas como resultado de experiências produzidas ao longo do tempo. Na percepção de Fleck, a enfermidade, a exemplo do beribéri, é apreendida como um evento humano e histórico. Sendo assim, sua compreensão social e cultural se modificou no Ocidente, à medida que a ciência avançava.

A terminologia médica “beribéri” advém do cingalês e está relacionada à fraqueza. É conhecida desde a Antiguidade no Japão e descrita também nos primeiros tratados médicos chineses. No Brasil, não existe um momento, propriamente dito, para datar a doença no território. Antes de ser diagnosticada, era referida, entre outras designações, como “inchação das pernas” e “perneiras”. Em 1648, o médico holandês Guilherme Piso (1957) registrou uma moléstia denominada “estupor dos membros”, semelhante ao beribéri. De acordo com Almeida (1916), em 1756 vigorava no Maranhão uma paralisia endêmica que remete, por analogia, ao mal. Na Corte, em 1786, tem-se notícia de uma epidemia reinante chamada de zamparina. Na capitania do Rio Negro, Antônio José Braga denominou-a *paralysia beriberium*.

No Brasil oitocentista, o “beribéri” manifestava-se endêmica e, às vezes, epidemicamente. Essa circunstância chamou a atenção dos médicos da época, que passaram a mapear sua difusão nas diversas regiões, constituindo-se uma geografia médica (Loureiro, 1885). A partir de 1866, tornou-se epidêmica na Bahia, conforme as investigações fomentadas pelo doutor José Francisco da Silva Lima, em cuja opinião, o beribéri, que acometia todos os indivíduos sem distinção social, era moléstia que se manifestava de forma endêmica ou epidêmica, caracterizada por dormência das extremidades, irritabilidade cutânea, fraqueza geral, entre outros sintomas. Os resultados dessa inquirição foram divulgados em vários números da revista *Gazeta Médica da Bahia* com o título “Contribuição para a história de uma moléstia que reina atualmente na Bahia sob a forma epidêmica e caracterizada por paralisia, edema e fraqueza geral”. Os artigos publicados no periódico baiano foram, posteriormente, reunidos por José Francisco da Silva Lima e publicados na obra *Ensaio sobre o beribéri no Brasil*. Lima (1872) considerou o evento epidêmico por assolar diversas partes da província, bem como todas as categorias sociais. Ele descreveu o processo clínico com grande riqueza de observações e qualificou o beribéri em três formas clínicas: paralítica, edematosa e mista. Por meio de uma criteriosa investigação, fundamentada na realização de autópsias em escravos encarcerados e sujeitos a exames policiais, constatou-se que a doença não parecia difundir-se por contágio ou infecção, e sim devido a condições higiênicas gerais desconhecidas (Lopes Filho, 1998). Almeida (1916) elogia o trabalho de Silva Lima e afirma que ele formulou as bases da teoria alimentar que passou a estar em voga no início do século XX.

Em Mato Grosso, conforme o visconde de Taunay (2011), o beribéri grassou por ocasião da Retirada da Laguna (1867), após o conflito com o Paraguai. Menciona que “continuava o beribéri a fazer em nossas fileiras numerosas vítimas naquele lugar ainda sujeito à influência dos grandes pantanais que a tropa acabara de atravessar, entre o Coxim e Miranda” (p.37). Para o nosso informante, tratava-se de uma epidemia climática de novo gênero, a paralisia reflexa, ou beribéri, que dizimou ainda mais soldados.

A memória intitulada “O beribéri na província de Minas Gerais”, datada de 1872 e elaborada pelo mineiro Antônio Felício dos Santos (1875), disserta sobre a incidência do achaque na região, que insurgiu pela primeira vez no Seminário de Nossa Senhora da Boa Morte de Mariana (1858-1861) e, posteriormente, avançou para o Colégio do Caraça^1^ (1861), Diamantina (1876) e Patrocínio (1876) (Azevedo, 1875). Santos realizou um criterioso exame em seus pacientes: incluiu informações como idade, observou a evolução da doença e em que época do ano ocorria, elencou ainda aspectos geográficos, ambientais e epidemiológicos para compor o cenário nosológico das ocorrências. De acordo com Lopes Filho (1998), ainda que Felício dos Santos e Silva Lima não afirmem categoricamente, em seus estudos, a ideia de um agente microbiano para o beribéri, essa opinião não era rejeitada, pois tais considerações preparavam o ambiente para divulgação da teoria microbiana.

O médico italiano Ignazio Aquiles Betoldi (1878) estudou o beribéri na província de São Paulo e confirma, com veemência, sua existência na região, ao contrário de vários dos seus colegas de profissão, que negaram, de acordo com ele, sua presença. Na sua concepção, “a grande maioria dos práticos da Província nega a existência dela, e diz que só existe na nossa imaginação desvairada, para qual todas as moléstias são beribéri” (p.3). Em relação a sua etiologia, afirma que é causada por um miasma, a exemplo de tifo, febre intermitente, febre amarela e todas as doenças infecciosas. Contudo, “é miasma *sui generis*, porque seus efeitos são também *sui generis*” (p.9). Adverte, também, “que o beribéri não pode ser filho do impaludismo, porque apresenta-se onde não há águas estagnadas, como são as altas e ventiladas montanhas de Minas Gerais” (p.11). Essa passagem é interessante porque mostra que o clínico tinha acesso à pesquisa realizada por Felício dos Santos em Minas Gerais. A relação do beribéri com a malária vigora até o início do século XX. Na Amazônia, entretanto, estudos de Carlos Chagas (1972) afastaram essa ideia, considerando que muitos casos da enfermidade eram um tipo de malária quartã ou uma variedade mais virulenta.

Virgílio de Mello Franco, em 1888, registrou a incidência do beribéri em Goiás, afirmando que se tratava de uma doença conhecida como “inchação de pernas” que acometia quase todo o Brasil, com mais intensidade, porém, nas províncias do norte e nos sertões de Goiás e Mato Grosso. Ele lamenta o atraso da medicina empírica em vigor no Sertão e a irracionalidade da terapêutica, sobretudo para o tratamento dessa moléstia, caracterizada, conforme sua impressão, por anemia profunda (Franco, 1888). Registros de óbitos emitidos pelo Hospital de Caridade São Pedro de Alcântara, da cidade de Goiás, confirmam que a mazela estava disseminada na região ao longo do século XIX (Magalhães, 2014b).

Em 1899, o médico alemão August Hirsch registrou a explosão desenfreada da doença no Brasil, sobretudo em Amazonas, Pará, Maranhão, Bahia, Espírito Santo, Rio de Janeiro, São Paulo, Paraná, Santa Catarina e Mato Grosso (Kiple, 1988).

A expansão populacional do Rio de Janeiro, Salvador, Recife, São Luís e São Paulo, nos séculos XVIII e XIX, evidenciou as mazelas sociais, promovendo estudos sobre a pobreza, as doenças e a deficiência alimentar (Magalhães, 2014b). As monografias médicas oitocentistas passaram a privilegiar as temáticas relacionadas a nutrição, habitação, costumes dos habitantes e saúde, observando a relação desses fatores com a ocorrência de males endêmicos e epidêmicos. Manuel da Gama Lobo (1865) relacionou a xeroftalmia (lesão da córnea) e a hemeralopia (cegueira noturna) entre os escravos a uma dieta pobre em nutrientes, composta por angu, abóbora e carne-seca ou toucinho, uma ou duas vezes por semana. Em *O regime alimentar no Norte do Brasil,* de 1881, Raimundo Nina Rodrigues indicou as deficiências oriundas da refeição restrita à farinha de mandioca.^2^ Em 1887, Hernani da Silva Pereira, formado na Faculdade de Medicina da Bahia, dissertou sobre a estratificação da dieta entre os mais abastados, que ingeriam carne fresca e feijão, e os mais pobres, que tinham uma comida monótona baseada em carne-seca, fubá e farinha (Vasconcelos, 2007).

É notório que os estudos realizados por médicos da Bahia, no campo da higiene alimentar, revelaram os primeiros relatos contundentes sobre casos de beribéri, muito antes da descoberta científica da vitamina B1.

Mesmo que os relatos médicos evidenciem que a doença estivesse disseminada pelo território brasileiro no Oitocentos, sua notificação para as autoridades competentes não era obrigatória. Fato que justifica, em parte, as raras informações relativas a algumas regiões.^3^ Assim, a legislação sanitária legitimava o conhecimento científico constituído, à medida que reconhecia a existência de um agente infeccioso determinante do beribéri (Lopes Filho, 1998). O regulamento sanitário de 1889 tornava-a de notificação facultativa (Brasil, 18 dez. 1889). Somente no decreto 5.156 de 1904 (Brasil, 8 mar. 1904) a enfermidade passou a ser de registro compulsório, o que contribuiu para a investigação mais criteriosa dos casos, a avaliação dos riscos aos quais as pessoas estavam sujeitas, a identificação da ocorrência por área geográfica e a definição das prioridades de intervenção.

Na Faculdade de Medicina da Bahia verifica-se um acentuado interesse pelo beribéri no período de 1877 a 1921, sobre o qual Meirelles et al. (2004) listam 19 teses que discutem a sua origem, mas sem chegar a um veredito. As temáticas alimentação e avitaminose também ganham espaço na instituição, com sete trabalhos defendidos. Todavia, a etiologia da moléstia permanecia misteriosa (Magalhães, 2014a). Contudo, o baiano Nina Rodrigues, em 1904, ao investigar casos de mortes no Asilo São João de Deus, formulou a hipótese de que as causas da doença reinante na instituição deveriam ser procuradas nas precárias condições de existência dos asilados, submetidos a maus-tratos e fome. Ele cogitou a etiologia carencial do mal, refutando a concepção miasmática e a do contágio (Jacobina, Carvalho, 2001).

A partir de 1880, médicos brasileiros e estrangeiros passaram a estudá-la intensivamente. K.C. Carter (1977, p.125) quantifica o notável interesse pelo tema a partir dos arrolamentos bibliográficos realizados por Henrich Botto Scheube e William Leonard Braddon:

A bibliografia quase completa de Scheube inclui duas publicações sobre o beribéri entre 1800 e 1809; nas décadas subsequentes as publicações totalizaram oito, dez, onze, trinta, sessenta e quatro, oitenta e, entre 1880 e 1889, cento e oitenta e uma. A bibliografia incompleta de W. Leonard Braddon lista quase duzentos artigos e livros da década de 1890 a 1899; outra bibliografia incompleta lista duzentas e cinquenta publicações para o período de 1900 a 1910. Uma bibliografia completa só para este período certamente se aproximaria de quinhentos itens.

Carter (1977, p.125) menciona que, decorridas três décadas, um editorial da revista *Lancet*, datado de 1911, evidencia que “provavelmente não existe doença sobre a qual houve tanta discussão quanto à sua etiologia como o beribéri”.

A revista *O Brasil Médico*^4^ constitui, também, uma fonte essencial para investigar o debate sobre a etiologia da moléstia de 1885 até meados da década de 1950, época em que a enfermidade deixa de ser o centro da atenção dos médicos agregados no periódico. Não obstante, os estudos sobre as vitaminas vão granjeando cada vez mais espaço na publicação. Somando os artigos publicados nos *Anais da Academia de Medicina do Rio de Janeiro*, período de 1885 a 1887, e os da revista *O Brasil Médico*, entre 1888 e 1942, tem-se 99 matérias sobre o tema.

**Figura 1:** : Gráfico com o número de matérias médicas sobre o beribéri na revista *O Brasil Médico*, 1888-1942 (fonte: elaborado por Sônia Maria Magalhães)

O arrolamento realizado no banco de obras da Biblioteca Nacional apurou 64 trabalhos que contemplam a moléstia. Ao se pesquisar a *Gazeta Médica da Bahia* (Meirelles et al., 2004), a revista *O Brasil Médico,* o acervo da Biblioteca Nacional e o acervo da Fiocruz, observa-se vigoroso e profícuo debate em torno do mote, em sintonia com as discussões que ocorriam em nível mundial.

Desde o século XIX, quando a etiologia do beribéri passou a ser intensivamente investigada, não havia consenso na medicina acerca da sua origem, de modo que suas formas de transmissão eram ignoradas. Nesse cenário, havia hipóteses admissíveis: a infecção provocada pelos miasmas mórbidos – emanações deletérias provenientes do meio ambiente – ou o contágio causado por um veneno específico e transmitido de pessoa para pessoa, por intermédio de objetos ou pelo ar respirado pelos enfermos (Chalhoub, 1996, p.64). Posteriormente, essas teorias foram renovadas, graças ao desenvolvimento científico e à teoria microbiana de Pasteur e Robert Koch, que associava várias doenças à ação de agentes microscópicos. Essas teorias inovadoras promoveram mudanças significativas nos procedimentos, métodos terapêuticos e profiláticos no tratamento das moléstias, contudo, “suas premissas penetraram lentamente o saber e a prática médica, assim como fomentaram várias disputas e controvérsias entre partidários e opositores desse novo paradigma” (Malaquias, 2016, p.736).

Como a teoria da deficiência alimentar parecia incompatível com os fatos, a maioria dos primeiros observadores preferiu outras explicações para a enfermidade. Talvez a mais comum das teorias alternativas fosse a de origem “miasmática” ou “malárica”. William Anderson, por exemplo, um dos primeiros escritores ocidentais a investigá-la no Japão, observou que a maior parte dos médicos japoneses acreditava que era causada por alguma emanação venenosa do solo. Assim, ele concluiu que a doença, provavelmente, explicava-se pela existência de um “veneno” atmosférico (Carter, 1977). Seguindo essa mesma tendência, o doutor Betoldi (1878) acreditava que a enfermidade que vigorava na província de São Paulo era causada por miasmas.

Do mesmo modo que existiam médicos que defendiam a teoria da infecção, havia outros que agregavam a suas concepções pesquisas recentes que vinculavam a origem da enfermidade à deficiência alimentar. Em 1889, o pesquisador holandês Christiaan Eijkman (1858-1930) isolou da cutícula do arroz uma substância solúvel na água e no álcool, a qual denominou princípio antineurítico que curava a polineurite das aves (Rezende, 2009). O cientista Gerrut Gerrit Grijns (1865-1944), também holandês, avançou na pesquisa e formulou a teoria de que a doença não era causada pela toxina, mas pela carência de uma substância existente na cutícula do arroz (Rezende, 2009).

Em 1910, várias linhas de trabalho convergiam rapidamente para a teoria relacionada à carência alimentar. De acordo com Carter (1977), os estudos epidemiológicos (Takaki, Voderman, Braddon, Fletcher, Stanton e Fraser) e os experimentos com animais (Eijkman, Grijns, Holst e Frolich) exibiram uma conexão entre certas dietas de grãos moídos e vários distúrbios. Os químicos (Eijkman, Fraser e Stanton, Funk), posteriormente, isolaram e caracterizaram com bastante precisão o ingrediente específico no polimento de arroz que preveniria e curaria alguns desses distúrbios.

O gráfico na Figura 1 mostra um avigoramento do debate médico na segunda década do século XX, sobretudo em 1916, graças à difusão da teoria das vitaminas, quando o químico polonês Casimir Funk,^5^ em 1911, identificou a química da vitamina, cunhando o termo após estudar a tiamina, posteriormente denominada vitamina B1. As descobertas do polonês impulsionaram um acalorado debate sobre o beribéri entre os médicos brasileiros. Em *O Brasil Médico* é possível perceber como as discussões em torno das vitaminas vão ganhando cada vez mais espaço.

No mesmo ano em que é possível verificar o aumento das publicações em *O Brasil Médico*, o médico mineiro Theophilo de Almeida, adepto da teoria alimentar, defende a tese *O beribéri no Brasil*. Nesse trabalho, ele critica o desinteresse dos colegas pela doença e enfatiza que:

Ultimamente irrompeu a longa discussão travada no seio da ‘Academia Nacional de Medicina’ e pela imprensa, reavivando as velhas teorias etiológicas, as múltiplas concepções patológicas, levantando uma verdadeira celeuma científica cujo grande fruto, se outros não colhesse, foi sem dúvida chamar para o assunto a atenção dos médicos brasileiros, que já iam, infelizmente, se esquecendo do seu ‘querido’ beribéri (Almeida, 1916, p.22).

Almeida (1916, p.22) ressalta serem dignos de menção apenas os estudos dos professores Antônio Austregilo Rodrigues Lima, Oscar de Souza, Henrique Duque e João Soledade, J. Silvado, Júlio Novaes, e Lídio Parahyba. O mineiro reflete, à luz do seu tempo, que a teoria do arroz descascado carecia de estudos mais aprofundados no Brasil.

### A alimentação do brasileiro

A partir da década de 1920, a relação do beribéri com a deficiência alimentar ganhava ampla difusão nas páginas dos principais jornais do país. O público leitor passava a ter cada vez mais acesso ao debate travado pelos cientistas brasileiros, graças ao processo de circulação, intercâmbios, contatos, distribuições, conexões, assimilações e ressignificações em torno da matéria. Segundo Ilana Löwy (2012, p.24), “os ‘fatos científicos’ são também enriquecidos pela sua circulação fora do domínio fechado da pesquisa científica. Fleck se interessa, assim, pelas interações entre representações científicas e populares dos fenômenos naturais”. Em relação ao processo de circulação, Kapil Raj (2015) adverte que o conceito de circulação ressalta as relações de poder, as resistências e as negociações inerentes às interações interculturais. Os estudos de Raj permitem observar a existência de comunidades médicas e científicas no Brasil que se orientaram para a resolução de problemas do país, colocando em questão o protagonismo de uma ciência pensada nos moldes estrangeiros, como os da Europa e dos EUA. Esse processo dinâmico conferia protagonismo a diferentes atores envolvidos no debate científico. Em entrevista de primeira página ao jornal *A Noite* de 23 de agosto de 1924, o médico José Paranhos Fontenelle critica a dieta alimentar do brasileiro composta por carne, feijão e arroz^6^ e evidencia a emergência da introdução de frutas, legumes e leite no repasto cotidiano. Ele faz notável referência ao desenvolvimento da ciência e a descoberta das vitaminas:

Outra importantíssima verificação moderna da ciência alimentar foi a demonstração da necessidade de certas substâncias indispensáveis à Nutrição para que o crescimento se perfaça e a saúde se mantenha, substâncias estas existentes em quantidades muito reduzidas, mas gozando do alto poder de estimular e regularizar a nutrição são as chamadas ‘vitaminas’ existentes em relativa concentração no leite, nos legumes, nas frutas e no óleo de fígado de bacalhau. Essas vitaminas já são classificadas em vários grupos e a falta de algumas delas é causa do beribéri, de escorbuto, de raquitismo, de mal crescimento, de anemia etc. (Fontenelle, 23 ago. 1924).

Em relação aos hábitos alimentares do brasileiro, o doutor ressalta o irrisório consumo de leite e adverte sobre a necessidade de inseri-lo na alimentação cotidiana. Esse assunto se tornou questão de Estado na década de 1930, quando houve investimento vigoroso na propaganda comercial do leite, devido à importância que a nova ciência da nutrição dedicava a esse alimento, mas havia empecilhos de diversas naturezas para sua difusão. De acordo com Brinkmann (2014) a questão do fornecimento do leite de vaca no Rio de Janeiro era muito difícil, pois não exigia somente a mudança cultural dos hábitos alimentares, mas também uma organização do sistema de abastecimento que excedia a capacidade de intervenção do estado brasileiro. Além disso, o leite, caro para a maioria da população, era um artigo de luxo. Fontenelle (23 ago. 1924) reserva um lugar especial para as mulheres, advertindo ser “preciso propagar intensamente o valor dos diversos alimentos e ensinar às donas de casa muitos meios de variar a alimentação, enriquecendo a cozinha brasileira de pratos em que figurem o leite e os legumes. Tudo deve ser feito para combater o sistema, carne-arroz-feijão, cujos elementos são excelentes, mas não podem bastar, só por si”.

Mesmo com a divulgação dos novos experimentos orientados para a etiologia alimentar da patologia, não havia se estabelecido um coletivo de pensamento médico sobre o beribéri no Brasil até meados do século XX. Esse coletivo, de acordo com Fleck, seria constituído a partir de uma comunidade na qual ocorreria influência recíproca de ideias, troca de saberes entre si, construindo um conhecimento orientado para a compreensão e mudança da realidade existente e resultante de uma atividade social, portanto, coletiva.

No Brasil da década de 1930, o problema da etiopatogenia do beribéri ainda dividia os médicos em duas categorias, a dos vitaministas e a dos que acreditam na origem infecciosa. O Quadro 1 sintetiza as vertentes médicas.

**Quadro 1:** : Médicos vinculados às teorias da infecção e das vitaminas do beribéri

Teoria infecciosa	Teoria vitaminista
Silva Lima, Torres Homem, Martins Costa, Pacífico Pereira, Francisco de Castro, Azevedo Sodré, Pedro de Almeida Magalhães, Sampaio Vianna, Miguel Couto, Clementino Fraga, Oscar de Souza, Eduardo Meirelles	Arlindo de Assis, Afrânio Peixoto, Hélio de Araújo Maia, Vicente Baptista, Figueiredo Rodrigues, Theophilo de Almeida

Fonte: Peregrino Jr. (1935)

É interessante observar que Peregrino Júnior recupera e valida estudos do século XIX, sugerindo que o beribéri pode ser causado por microrganismos encontrados nos pacientes (Almeida, 1916, p.21-22). Pacífico Pereira encontrou alguns corpúsculos redondos no sangue de doentes, na Bahia. Augusto Maia descobriu um tipo de micrococcus. Paulo Mendes observou alguns germes na medula. Em 1883, João Batista de Lacerda deparou-se com um bacilo que considerou ser a causa da doença. Francisco Fajardo detectou, em 1898, um hematozoário semelhante ao da malária (Almeida, 1916). Carter (1977) enfatiza que a teoria dos germes influenciou muitos cientistas e fomentou estudos demográficos e experimentos químicos em animais que forneceram um novo padrão para a compreensão teórica da doença. Destarte, a ciência avançava à medida que hipóteses – verdadeiras e falsas – eram testadas experimentalmente.

Em trabalho de 1913, “Notas sobre epidemiologia do Amazonas”, Carlos Chagas comenta que as intensas epidemias de beribéri e os muitos casos de polinevrites (múltiplas inflamações dos nervos) no Acre se deviam a um diagnóstico que teria como elemento comum o edema, sem que os doentes apresentassem as perturbações características da moléstia, que representariam “modalidades da malária”. O clínico confirma a raridade dos casos e faz referência a uma condição mórbida especial, o chamado “beribéri galopante”, caracterizado por edema progressivo que se iniciava nos membros inferiores e alastrava pelo tronco, levando o enfermo a óbito em curto prazo de tempo (Chagas, 1972, p.167).

Afrânio Peixoto (1972), por sua vez, atestou que o beribéri na Amazônia se justificava pela insuficiência alimentar, por alimentos pobres em vitaminas, principalmente pela farinha d’água. Recomendou que, para preveni-lo e tratá-lo, era essencial um nutrimento adequado, incluindo ovos, peixe, feijão, frutas e legumes frescos.

Theophilo de Almeida (1916, p.19) destaca a repercussão dos estudos relacionados à teoria alimentar: “Cresceu o prestígio do arroz na etiologia do beribéri, apesar das contraditas, que as tem tido até no próprio Oriente, o Congresso Internacional de Medicina realizado em Londres, em 1913, adaptando a proposta de Braddon^7^ resolve o seguinte: reconhece que a alimentação básica pelo uso contínuo do grão sob a forma de arroz branco produz beribéri”.

O Congresso Internacional de Medicina Tropical, a despeito de partidários contrários, confirmou a teoria alimentar fundamentada no consumo do arroz branco pilado, comprovando que o beribéri não era uma infecção. A declaração de Londres referendava, praticamente, a hipótese do doutor inglês Willian Braddon que observou a origem patogênica da doença, atribuída a um “veneno” (produzida por um organismo epífita ou parasita) presente no arroz (Almeida, 1916). Nesse sentido, o evento recomendou a extinção de quarentenas. Sobre a admissão da teoria alimentar, Almeida afirma que os delegados brasileiros, os médicos Juliano Moreira e Ernesto Crissiuma, não tomaram parte da discussão.

Em entrevista ao jornal *A Noite*, Crissiuma descreve que:

Em relação ao beribéri foi também votada uma outra ordem do dia pedindo que se abolisse a quarentena dos navios que vêm de países infeccionados por esta moléstia, por não ser o beribéri considerado contagioso e ao mesmo tempo a proibição do arroz branco como base da alimentação das classes trabalhadoras, especialmente os *collies*, que exercem a sua atividade na Índia e na China (Entrevista..., 6 dez. 1913).

Crissiuma admitiu que o congresso, que recebera em torno de oito mil participantes, proporcionou grandes resultados: “Desta vez posso assegurar-lhe, os congressistas trabalharam a sério as mais palpitantes questões que mais de perto interessam a ciência em cada um dos países ali representados” (Entrevista..., 6 dez. 1913). Além do beribéri, a sífilis, a lepra e a leishmaniose foram amplamente discutidas, destacando-se nos debates clínicos da França, da Inglaterra e da Alemanha. Afora Moreira e Crissiuma, a delegação brasileira foi composta pelos médicos Clementino Fraga, Marcos Cavalcanti, Henrique Roxo e Rocha Lima.

A teoria do arroz polido como causa do beribéri gerou polêmica entre médicos brasileiros, uma vez que ela não explicava sua incidência no país, pois o cereal não era item importante na dieta, com poucas exceções em algumas áreas do Nordeste. A carne fresca de vaca existia, porém, era inabitual e cara. No Rio de Janeiro e em São Paulo, por outro lado, a dieta dos pobres centrava-se exclusivamente na carne-seca. O processo de secagem da carne-seca – que consistia, primeiramente, em salgar e depois colocá-la para secar ao sol por no mínimo dois meses – destruía a tiamina contida no alimento. Como a substância é altamente solúvel em água, grande parte era descartada no processo de dessalgação da proteína. A tiamina restante, sensível ao calor da água, era destruída no processo de cocção prolongado^8^ (Kiple, 1988).

A grande vilã da dieta do brasileiro, no entanto, era a farinha de mandioca, que tem ainda menos tiamina do que o arroz polido. No Nordeste, onde a comida da maioria daquela gente era fundamentada na farinha de mandioca, o beribéri era mais nocivo. A mesma situação era observada entre os pobres de São Paulo e Rio de Janeiro, que centravam sua dieta na carne-seca, sendo, portanto, as maiores vítimas. Na percepção de Kiple (1988), a teoria alimentar se justificava no Brasil devido à combinação nefasta entre carne-seca e farinha de mandioca. Somente no século XX, graças à emergente ciência da nutrição, descobriu-se que a enfermidade era uma hipovitaminose.

Ainda na década de 1930 não havia unanimidade entre os médicos acerca do beribéri como uma avitaminose. Em uma matéria intitulada “Vitaminas e avitaminoses”, publicada na revista *O Brasil Médico* de 1935, Oscar Ferreira Júnior, assistente da primeira Cadeira de Clínica Médica do Professor Oswaldo de Oliveira, menciona: “Ora o problema das vitaminas na etiologia da enfermidade não está de modo algum resolvido; o problema continua complexo e insolúvel” (Ferreira Jr., 20 jul. 1935, p.654).

Os pesquisadores brasileiros, defendendo as teorias infecciosa e alimentar, esforçaram-se para delinear o beribéri e seus sintomas, atribuindo a ele um quadro clínico. O debate em torno de suas causas e origem resultou no estabelecimento de um campo médico entre os profissionais dedicados à compreensão do mal. Nesse sentido, as representações médicas ganharam relevância social à medida que resultados de pesquisas eram divulgados em editoriais de jornais, congressos e outros meios de difusão, descrevendo os sintomas, percepções dos pesquisadores e narrativas científicas que se formavam em torno do enfermo. Esse processo, entretanto, foi marcado por controvérsias no meio médico. Por causa da circunscrição da medicina da época e da dificuldade de a distinguir clinicamente, e antes da existência de técnicas laboratoriais e da popularização dos microscópios, o diagnóstico gerava impasses mesmo entre os médicos. Fato é que, em razão do conhecimento restrito sobre as formas de manifestação da doença, sua prevalência em algumas regiões e seus desconhecimento em outras, muitos diagnósticos eram imprecisos e, frequentemente, equivocados.

As contribuições das pesquisas realizadas geraram profícuos debates em torno do beribéri e aportes primordiais para a construção de questões a responder. Como menciona Fleck (2010, p.153),

cada fato repercute retroativamente em outros, e cada mudança, cada descoberta exercem um efeito em um campo que, na verdade, não tem limites: um saber desenvolvido, elaborado na forma de um sistema harmonioso, possui a característica de cada fato novo alterar todos os anteriores, por menor que seja essa alteração. Nesse caso, cada descoberta é, na verdade, a recriação do mundo inteiro de um coletivo de pensamento.

As diversas linhas de pesquisa, relativamente independentes, constituem a base para endossar a teoria carencial. Era necessário, contudo, organizá-las e lhes conceder coerência. A primeira publicação em que se reuniram as variadas vertentes foi elaborada em 1912 por Casimir Funk em “The etiology of the deficiency diseases”, quando ele apresentou uma substância nova que era essencial para a manutenção da vida: a “vitamina” (Carter, 1977).

Compreende-se que estava em curso o processo, apresentado por Fleck, como conformação de um estilo de pensamento. O beribéri concedia espaço a uma nova interpretação no campo médico, deixando gradativamente de ser admitido como uma doença infecciosa e passando a ser compreendido sob uma nova perspectiva, a alimentar, que era compartilhada entre os pesquisadores, embora tenha demorado décadas para seu reconhecimento. Isso se constituía como um fato médico-científico, uma vez que era assimilado e expresso no coletivo de pensamento que estava sendo construído na época.

### As vitaminas no centro do debate

Com a difusão da teoria vitamínica, a inadequação da dieta nacional – carente em leite, laticínios, ovos, verduras e frutas – foi colocada na pauta do dia, no momento em que a nutrição alcançava o estatuto de ciência e os conhecimentos sobre a composição dos alimentos e sobre a analogia entre dieta e enfermidade (Magalhães, 2014b, p.44):

Sob a influência dos avanços da bioquímica, que propiciaram uma melhor compreensão do funcionamento do organismo humano e a identificação de novas substâncias, como as vitaminas, o mote passa a ser investigado, abordado ou questionado por vários estudiosos em diversas áreas do conhecimento.

É no interior desse contexto de transformações sociais, entre as décadas de 1930 e 1940, que os médicos nutrólogos Josué de Castro e Nelson Chaves produziram relevantes estudos sobre a alimentação brasileira, “justamente num período em que a questão da fome mobilizava vários setores da sociedade brasileira em decorrência da inflação do custo de vida provocada pela crise de 1930” (Magalhães, 2014b, p.44). De acordo com Leme (2021, p.1127), “a nova perspectiva científica, portanto, forneceu uma ferramenta central para que Castro e seus pares pudessem olhar para a questão alimentar no Brasil – o paradigma calórico”.

Em 1932, Castro realizou um inquérito^9^ alimentar pioneiro sobre as condições de vida dos operários no Recife, divulgando a péssima qualidade da alimentação desse segmento, marcada pela insuficiente ingestão calórica. As pesquisas para quantificar e avaliar o nutrimento das classes populares conquistaram cada vez mais espaço na Europa no começo do século XX, uma vez que a alimentação passou a ser passível de medição (Leme, 2021). O grande mal dos trabalhadores do Nordeste era revelado pelo médico. Era a fome que os impossibilitava de acompanhar o ritmo dos trabalhadores do sul do país, ou mesmo da zona do sertão, identificadas por ele como regiões de melhor nutrição. Suas pesquisas responsabilizaram a fome crônica, que acometia há séculos aquela gente, por sua incapacidade, bem como pela maioria das doenças e das mortes.

Em *Geografia da fome*, Castro (1948) elaborou um mapa das carências alimentares existentes nas diferentes regiões do Brasil, nomeadas por ele de Amazônica, Nordeste Açucareiro, Sertão Nordestino, Centro Oeste e Extremo Sul.

**Figura 2:** : Mapa das principais carências existentes nas diferentes áreas alimentares do Brasil (fonte: Castro, 1948, figura 6)

Das cinco áreas analisadas por Castro, três foram consideradas nitidamente de fome: ”Amazônica”, “Nordeste Açucareiro” e “Sertão Nordestino”. As do Centro Oeste e do Extremo Sul, por sua vez, não padeciam de deficiências alimentares tão notáveis, mesmo que a dieta não fosse a desejável. No mapa de Castro é possível perceber como o beribéri, carência de vitamina B1, a partir da configuração regional atual, estava disseminado no Norte e no Nordeste, com menos impacto, no Sudeste e no Sul do Brasil. Na opinião de Kiple (1988), o beribéri se apresentava com maior intensidade na região nordestina onde o regime alimentar básico, consistindo de mistura de carne-seca e farinha de mandioca, era deficiente em vitamina B. Na compreensão de Araújo (2022) as doenças carenciais no Rio Grande do Norte eram oriundas das relações desiguais entre as oligarquias proprietárias de latifúndios e a população que trabalhava nessas propriedades. É importante observar que Castro ratificou a teoria alimentar do beribéri brasileiro que encontrava sua justificativa no repasto baseado no binômio carne-seca e farinha de mandioca. No parecer de Kiple (1988), em Minas Gerais e no Rio Grande do Sul, ao contrário, a dieta se apresentava mais “uniforme” entre ricos e pobres. Entre os mineiros, a carne de porco fresca, o feijão e a farinha de milho estavam presentes no repasto de todos os grupos sociais. Se, por um lado, o milho poderia causar um desequilíbrio vitamínico pela deficiência da tiamina, por outro, a carne fresca de porco, rica em niacina, compensava o teor necessário de vitamina B. Quanto aos sulistas, notava-se na sua refeição o consumo de carne fresca de vaca, e de cereais e vegetais. O fator interessante é que os sulistas produziam a carne-seca (o charque), mas não a consumiam habitualmente. A produção era vendida aos nordestinos.

Castro e Chaves reafirmam a ênfase da alimentação racional sobre a dimensão racial/climática, buscando explicação para a qualidade de vida do brasileiro. Em *Alimentação e saúde pública*, Chaves (1948) responsabiliza a subalimentação pela elevada taxa de mortalidade infantil, natimortalidade, cárie infantil, retardamento do crescimento, estados carenciais, beribéri, pelagra e incidência das verminoses. Os elementos da denominada teoria do círculo vicioso da pobreza/doença, identificados nas obras de outros autores da época, também estão presentes nos estudos do autor: “O homem produz pouco porque se alimenta mal e é doente e, pelo seu nível educacional, tem um âmbito de aspirações muito reduzido e, produzindo pouco, não dispõe de quantidade suficiente de alimentos” (citado em Vasconcelos, 2001, p.329). Os pernambucanos atentaram para a problemática da monocultura e suas repercussões, especialmente no que tange à nutrição da gente da Zona da Mata nordestina. No parecer de Castro, a farinha com feijão, o charque e o café com açúcar perdiam, gradativamente, espaço na mesa do agricultor. A farinha e o feijão ralo, quando muito, compunham o sustento diário. As investigações de Chaves (1946, 1948) chamaram a atenção das autoridades para a grande incidência de nanismo na Zona da Mata de Pernambuco. Na sua percepção, a estatura e até o cérebro estariam diminuindo gradualmente pela fome. Por intermédio das temáticas fome e eugenia, os cientistas pernambucanos avigoraram a tarefa de construção da nacionalidade brasileira, uma vez que buscaram descentralizar suas análises da questão racial para a sociocultural, o preconceito racial/climático que se tinha em relação à mestiçagem (Magalhães, 2014b). Lima (2008, p.265) reforça esses argumentos e afirma que “o que uniu os intelectuais em torno da demonstração da tese de mal de fome no período de 1934 a 1939 foi a perspectiva de aperfeiçoamento de eugenia como fator de evolução”.

Naquele momento, as carências alimentares do Brasil já eram tema consagrado pela imprensa e por especialistas. Castro e Chaves conquistaram papéis de destaque nos estudos nacionais e internacionais sobre alimentação, fome, pobreza e nutrição. A partir dessa época, esses médicos nutrólogos, atuando como docentes e pesquisadores na Faculdade de Medicina do Recife, encontraram subsídios para legitimação e difusão das teorias alimentares, associando-as às políticas de Estado. Chaves ocupou os cargos de diretor-geral do Departamento de Saúde Pública e secretário de Saúde de Pernambuco. Castro, por sua vez, se engajou na primeira campanha das vitaminas realizada no Brasil e amplamente divulgada em *A Noite*: “Conforme já foi divulgado pela imprensa, a coordenação da Mobilização Econômica resolveu lançar a Campanha Nacional de Vitaminas que terá como finalidade prover a população brasileira desses elementos, tão necessários à vida. O certame será orientado por uma comissão diretiva, presidida pelo ministro João Alberto, e que tem como membros os srs Josué de Castro, chefe do Serviço Técnico da Alimentação C.M.E.” (A Noite, 14 jan. 1944). Assim, no período de 1934 a 1939, o Estado Novo, por meio de ações políticas e pedagógicas de educação alimentar, criou bases para ensinar o brasileiro a comer bem e a economizar (Lima, 2008).

O doutor Theóphilo de Almeida acompanhou, mesmo que indiretamente, o processo de reconhecimento da teoria alimentar do beribéri por parte das ações do governo, assumindo cargos públicos. Em 1923, foi nomeado médico do Departamento Nacional de Saúde Pública, dirigido por Carlos Chagas. Em 1941, década em que se inicia a campanha das vitaminas, tornou-se diretor da Divisão de Organização Hospitalar do Ministério da Saúde (Lopes Filho, 1998).

No percurso para desvendar a etiologia do beribéri e as formas de controlá-lo foi fundamental a atuação do coletivo de pesquisadores formado principalmente por médicos nutrólogos, vinculados a projetos de Estado. Esses profissionais especializaram-se nas suas pesquisas, o que possibilitou constituir um seleto campo “esotérico”, responsável por desenvolver o coletivo de pensamento em torno da enfermidade. A compreensão do beribéri, no entanto, percorreu outros domínios, pois em torno desse grupo se desenvolveu um amplo campo do conhecimento, composto por não especialistas, identificados por Ludwig Fleck como “exotérico” por meio de vários meios de imprensa, a exemplo dos jornalistas do tabloide *A Noite*, que assumiram o papel de divulgadores do conhecimento produzido pelos especialistas. Assim, ao apregoarem tais ideias, os não especialistas ratificavam sua confiança nas orientações advindas do coletivo “esotérico”, o que validava e expandia suas ideias, ficando clara a interdependência desses campos para a legitimidade social da ciência (Fleck, 2010, p.26). Ao simplificar o discurso clínico, torná-lo acessível ao público e demonstrar o impacto da doença na saúde da população, eles certificam o discurso “esotérico” ao propalar suas concepções sobre a doença.

O bem-sucedido trabalho pedagógico feito pelo coletivo dos “exotéricos” pode ser apurado, como exemplo, em *A Noite* do dia 27 de março de 1953 na coluna “Pondo os ‘pontos nos ii’”. Trata-se de um jogo recreativo de perguntas e respostas para ser praticado com amigos. São duas colunas, uma contendo a pergunta e outra, a resposta. A quarta pergunta é a seguinte: “Comer arroz causa a doença chamada beribéri?”. E a resposta: “Beribéri não é causada pelo fato de comer arroz, mas pela dieta desequilibrada ou falta de vitamina B, o que acontece com a alimentação exclusiva de arroz polido” (A Noite, 27 mar. 1953, p.7).

Ao estudar a enfermidade por décadas sem desvendar sua etiologia, os pesquisadores passaram a desenvolver inquéritos alimentares que permitiram construir narrativas no campo científico, descrevendo a geografia da doença, as condições de vida da população, características biológicas e socioculturais, possibilidades alimentares, consolidando assim uma forma de concebê-la, bem como outras avitaminoses, no campo médico-científico. Contudo, Castro não foi o único a fazer inquéritos alimentares. Leme (2021) menciona a pesquisa promovida pelo Instituto de Higiene de São Paulo, publicada em 1935, realizada por Geraldo Horácio de Paula Souza em parceria com Ulhôa Cintra e Pedro Egídio de Carvalho. Esses inquéritos, de acordo com a interpretação de Fleck, consolidavam um estilo de pensamento. Por intermédio da difusão dessas obras, as ideias nelas contidas passaram a circular entre os especialistas e os leigos esclarecidos*,* já que tais concepções transitavam em associações, congressos médicos, eventos científicos, entrevistas e editoriais de jornais, unificando as percepções desses médicos. Essas ações promoviam a disseminação e a concepção da forma de se pensar o beribéri naquele contexto histórico que, incluso no campo médico, ora eram defendidas, ora eram reelaboradas.

## Considerações finais

As controvérsias sobre o beribéri e a teoria microbiana, no final do século XIX, e carencial, no início do século XX, ensejam acompanhar o processo de institucionalização da ciência no Brasil. A teoria dos germes forneceu um novo padrão para a compreensão do beribéri relacionado à deficiência alimentar. Contudo, o processo de construção de um coletivo de pensamento médico, conforme a percepção de Fleck, admitindo a deficiência de vitamina B1, foi longo e repleto de embates, uma vez que as concepções sobre a etiologia da moléstia eram bastante diversas. Considerava-se sua predominância em sociedades que tinham o arroz como alimento de base, e sua incidência poderia estar relacionada à deterioração ou contaminação do cereal. Poderia haver conexão com a presença de vermes intestinais em alguns enfermos, sugerindo que os parasitas poderiam ser a causa do mal. Admitia-se também sua proeminência em ambientes específicos, tais como prisões, hospitais, colégios, quartéis, abrigos e navios, afetando muitas pessoas simultaneamente, indicando algum tipo de contágio. A partir da década de 1880, vários autores (no Brasil e no exterior) começaram a cogitar a causa por microrganismos. No início do século XX, uma nova proposição foi desenvolvida: a da carência alimentar. Casimir Funk afirmou que o beribéri era causado por carências combinadas de vários nutrientes. No Brasil, as teorias dos microrganismos e das vitaminas fomentaram um intenso e longo debate entre os médicos, que adentrou o século XX, até a efetivação de um coletivo de pensamento, reconhecendo que a patologia era causada pela deficiência de vitaminas, à medida que pesquisas subsequentes admitiram a maioria dos argumentos científicos formulados por Funk.

No século XXI, o beribéri volta a fazer parte da pauta médica no Brasil. Em 2007, 47 pessoas faleceram no Maranhão. Na percepção da medicina, os casos correspondem a um surto de uma enfermidade que já estava controlada. Em 2008, foram identificados casos suspeitos da moléstia em indígenas das etnias ingaricó e macuxi, no município de Uiramutã, no estado de Roraima, e, desde então, estão sendo empreendidas ações em parceria com o estado e com o município para inquirição, acompanhamento, prevenção e controle da doença. Em 2012, novos casos entre a população mais pobre foram registrados no norte do país. Esses episódios revelados na contemporaneidade fomentaram a organização do *Guia de consulta para vigilância epidemiológica, assistência e atenção nutricional dos casos de beribéri*, organizado pelo Ministério da Saúde em 2012 (Brasil, 2012). Trata-se de orientações para os profissionais de saúde de toda a rede de atenção do Sistema Único de Saúde (SUS) e de seu Subsistema de Atenção à Saúde Indígena e visa à adoção das medidas de detecção, prevenção e controle da moléstia. No período de 2017 a 2020, em virtude da crise econômica instalada e do crescimento da fome e da pobreza, acentuada pela pandemia da covid-19, houve aumento de registro de óbitos por beribéri. Santos e colegas (2023) conjecturam que o número de registros de óbitos por beribéri em 2021 e 2022 continuou em tendência de alta, em decorrência do agravamento da crise social e econômica. Os resultados de pesquisas, realizadas durante a pandemia, demonstram que a insegurança alimentar aumentou ainda mais no país, sobretudo entre os mais pobres, que já possuíam outras carências. Por ser uma enfermidade negligenciada e associada à pobreza, o beribéri não consta na lista de notificação compulsória do Sistema de Informação de Agravos de Notificação, colaborando para a subnotificação dos casos e óbitos e contribuindo para a invisibilidade da doença, dos doentes e suas implicações sociais. O Instituto Brasileiro de Geografia e Estatística (IBGE) apontou que a fome, que abre brechas para graves doenças nutricionais, esteve presente em 3,2 milhões de residências brasileiras em 2023. A escassez afetou não só os adultos, mas também crianças e adolescentes residentes nos lares atingidos pela fome. O IBGE definiu essa situação como insegurança alimentar grave (Ferreira, 25 abr. 2024).

Refletir sobre a historicidade e os impactos do beribéri no Brasil, ao longo do tempo até a atualidade, é de suma importância para a compreensão de como o homem e as instituições agiram diante da crise instalada, como encontraram políticas públicas de saúde e as promoveram para enfrentar e controlar o mal.

## Data Availability

Não estão em repositório.

## NOTAS

1 De acordo com Lopes Filho (1998), as mais cruéis epidemias do Caraça foram, além daquela ocorrida em 1862, as de 1873, 1878, 1901, 1906 e 1910. Em 1930, o beribéri é considerado extinto nessa instituição.

2
Carvalho (2025) mostra que este estudo foi divulgado no jornal *Pacotilha*, Maranhão, 1888, ano 8, nos números 154, 157, 158, 164, 167 e 188.

3 A legislação sanitária de 1889 tornava o beribéri doença de notificação facultativa. Esse decreto elenca como obrigatórios a vacinação antivariólica nos primeiros seis meses de vida, a desinfecção de locais e objetos contaminados, o isolamento hospitalar quando o doente não estivesse em condições de receber tratamento em casa e a notificação de doenças transmissíveis. De comunicação compulsória a febre amarela, a cólera, a peste, a difteria, a varíola, a escarlatina e o sarampo. E de notificação facultativa a tuberculose, a febre tifoide, a coqueluche e o beribéri. Somente no decreto de 1904, o beribéri passa para o grupo de comunicação compulsória.

4 A revista *O Brasil Médico* era um órgão semanal de divulgação de interesse científico, político, prático e profissional da classe médica brasileira, fundada em 1887 e editada até 1956, na cidade do Rio de Janeiro.

5 O polonês Casimir Funk nasceu em Varsóvia, em 1884. Em 1904, doutorou-se em química na Universidade de Berna. Trabalhou como bioquímico no Instituto Pasteur, no Hospital Municipal de Wiesbaden, na Universidade de Berlim e no Instituto Lister. Prosseguiu seus estudos em Paris e Berlim. Imigrou para os EUA em 1915. Trabalhou em laboratórios de Londres, Nova York, Paris, Varsóvia. De 1923 a 1927, Funk foi chefe do Instituto Nacional de Higiene da Polônia. De volta aos EUA, assumiu a presidência da Funk Foundation for Medical Research. Faleceu em Nova York, em 1967. Funk é reconhecido como um dos primeiros cientistas a formular o conceito de vitaminas. O Instituto Polonês de Artes e Ciências da América concede anualmente o Prêmio Casimir Funk de Ciências Naturais a cientistas polaco-americanos que se destacam no campo da ciência. Para saber mais sobre Funk, ler Griminger (1972).

6 Certamente Paranhos está discutindo a dieta dos grupos mais abastados, pois na alimentação dos pobres persistia a combinação carne-seca e farinha de mandioca, como bem enfatizou o médico Hernani da Silva Pereira em 1887 (Vasconcelos, 2001).

7 [Nota de Almeida (1916)] De acordo com Braddon, nos lugares em que é endêmico, ou durante uma epidemia, só são acometidos por beribéri os que comem arroz.

8 Na concepção de Kiple, o complexo B deve estar em equilíbrio no organismo humano. Cabe mencionar que a luz solar também destrói a riboflavina; sendo assim, a carne-seca tem fornecimento insuficiente de tiamina e riboflavina em relação à niacina, e, portanto, aumentaria consideravelmente as necessidades normais de vitamina B do indivíduo que a ingeria.

9 Para Leme, os inquéritos higienistas que pretendiam observar os hábitos dos brasileiros começaram a surgir no Brasil no início do século XX, mas sem pretensões de estatísticas. Arthur Neiva e Belisário Penna, em 1912, como exemplo, pesquisaram as condições de saúde da população pelos sertões de Pernambuco, Piauí e Goiás.
